# Evaluating Australia's “MyMedicare” voluntary patient registration system: the prospective evaluation of patient registration study protocol

**DOI:** 10.3389/frhs.2026.1746024

**Published:** 2026-03-19

**Authors:** Reema Harrison, Ashfaq Chauhan, Rebecca Mitchell, Smriti Raichland, Gaston Arnolda, Jeffrey Braithwaite, Johanna I. Westbrook, Elizabeth Manias, Janani Mahadeva, Bronwyn Newman, Ramya Walsan, Kate Churruca, Sam Ricketts, Ricki Spencer, Mashreka Sarwar, Jeffrey Liang, Deborah Pallavicini, Dalal Dawood Baumgartner, Kim Bowen, Donna Gillies, Charbel Badr, Konrad Kangru, Ai-Vee Chua, Louise Hardy, Sarah Judd-Lamm, Kirsten Moore, Lily Edwards, Sanjyot Vagholkar

**Affiliations:** 1 Faculty of Medicine Health and Human Sciences, Macquarie University, Sydney, NSW, Australia; 2 Faculty of Nursing and Midwifery, Monash University, Melbourne, VIC, Australia; 3 Sydney North Health Network, Chatswood, NSW, Australia; 4 SATB2 Connect, Sydney, NSW, Australia; 5 NDIS Quality and Safeguards Commission, Sydney, NSW, Australia; 6 Hunters Hill Medical Practice, Sydney, NSW, Australia; 7 Queensland Primary Health Network, Queensland, QLD, Australia; 8 Dubbo Family Medical Practice, Dubbo, NSW, Australia; 9 Arthritis Australia, Broadway, NSW, Australia; 10 Carers New South Wales, Sydney, NSW, Australia; 11 Australian Primary Health Care Nurses Association, Melbourne, VIC, Australia; 12 Royal Australian College of General Practitioners, East Melbourne, VIC, Australia

**Keywords:** general practice, patient enrolment, patient registration, person-centred care, primary care, voluntary patient registration

## Abstract

**Background:**

Managing the burden of chronic and complex disease is a global priority for health service delivery. Initiatives that aim to improve care integration and continuity to optimise health care utilisation, efficiency and outcomes are a priority. Patient registration with a general practice or practitioner has been adopted in multiple countries to promote continuity of care. This program of work will provide novel, critical evidence of the implementation of the Australian “MyMedicare” voluntary patient registration scheme, and the associated outcomes within its first years.

**Methods:**

Three workstreams will address five research objectives. Methods comprise analyses of longitudinal observational administrative health data (Workstream 1), qualitative interviews and quantitative surveys (Workstream 2) and analysis of linked general practice and health administrative data (Workstream 3). Primary outcomes are the number and demographics of patients registered in the MyMedicare scheme, implementation outcomes including feasibility and acceptability of registering, cost to practices associated with registering patients, and early impacts in the extent to which registration is associated with changes in health service utilisation, health outcomes and continuity of care.

**Conclusions:**

By evaluating over five years, the proposed research provides ample opportunity for people to have registered in order to assess, the process of registration, the effects of MyMedicare incentives, and the scheme's impact on continuity of care and health outcomes. The resulting evidence will contribute to national policy and international literature on the application of patient registration models to promote health service delivery and outcomes in the context of an aging population with escalating chronic and complex disease burdens.

## Background

An escalating chronic disease burden is impacting the delivery of health services internationally. With the estimated cost of chronic disease expected to reach USD$47 trillion worldwide by 2030, reforms throughout health systems globally have sought to ensure sustainable and effective provision of health services (1). In Australia, almost half of the population (47%) has chronic or complex health conditions that require two or more healthcare professionals or services to collaborate in providing optimal, holistic care (2, 3). Central to these health system reforms are initiatives that aim to improve care integration and continuity to optimise health care utilisation, efficiency and outcomes for people with chronic or complex health conditions (4–7).

Continuity of care (CoC) describes “*the degree to which a series of discrete health care events is experienced by people as coherent and interconnected over time and consistent with their health needs and preferences*” (8). CoC is often conceptualised in three domains; relational, informational and managemental (9). Relational CoC refers to the ongoing therapeutic relationship between patients and their care providers. In primary care settings this may include a continued therapeutic relationship between patients and one or more general practitioners (GPs), registrars, nurses, allied health or care coordinators. Providers’ knowledge of the person is identified as an important mechanism to provide them appropriate care. Informational CoC describes the access to, and use of, patient's health and social information by GPs to ensure that appropriate care is provided. Managemental CoC then addresses consistency and coherence in patient care provision across changing health status. All domains of CoC can greatly improve the quality of care for individuals, particularly those managing two or more health conditions (10). In addition to improving care outcomes, CoC promote positive care experiences, whilst maximising cost-effectiveness (10).

Internationally, patient registration with a GP or medical practice has sought to facilitate CoC by formally linking patients to their primary care providers (11). CoC may be achieved by promoting the relationship between registered patients and their preferred providers or practices, coordination of care by the preferred provider, and supporting the exchange of relevant information via the registered practice. Patient registration has been adopted in several countries, with characteristics of patient registration models determined by the health system context (12, 13). In the United Kingdom (UK), through the National Health Service (NHS), mandatory patient registration with an individual GP or practice was introduced in 1948. Mandatory patient registration was adopted to enable capitation payment to GPs who are responsible for care and referrals of their cohort of patients (14). In Canada, voluntary patient registration (VPR) was introduced in Ontario in 2001 to provide a basis for remuneration of GPs for a group of patients based on capitation *or* salary. More recently, Singapore introduced VPR in 2023 through the Healthier SG scheme, with a strong focus on preventive health and developing an integrated health and social ecosystem (15).

### Patient registration in the Australian health system

Medicare, established in 1984, is Australia's national health insurance scheme providing free or subsidised healthcare to Australian and New Zealand citizens, permanent residents, and visitors covered under the Reciprocal Health Care Agreement. In the 2023–24 Federal Budget, the Australian Government introduced VPR under the MyMedicare scheme (16). All individuals eligible for Medicare can register for MyMedicare. Patient registration is one component of a broader reform agenda outlined in Australia's Primary Health Care 10-Year Plan (2022–2032) (17), which addresses challenges posed by an ageing population and rising rates of chronic and complex conditions. The scheme represents the fifth major strategic initiative since the 1990s that has aimed to improve CoC through primary care reform. The MyMedicare scheme has received substantial investment: AUD $19.7 million over four years for implementation and an additional AUD $39.8 million to support delivery. Incentives for patients and providers (Box 1) have been introduced progressively since MyMedicare's official launch on 1 October 2023. Further funding is anticipated for frequent hospital service users, tied to regular GP visits.

Box 1 **Released MyMedicare Incentives 2023–2025.**

**Table:** 

October 2023: Registered patients with chronic conditions and aged care residents gained access to additional Medicare Benefits Schedule (MBS) items (billing numbers).
November 2023: Eligible groups, including children under 16, pensioners, concession card holders, and patients with complex needs, able to access longer subsidised telehealth consultations.
July 2024: The General Practice in Aged Care Incentive (GPACI) restructured funding to support registered GPs and practices providing care in Residential Aged Care Homes (RACH).
July 2025: Reformed Chronic Condition Management items introduced for registered patients, linked to care by their nominated practice.

### Implementation risks and equity challenges

For MyMedicare to achieve its objectives, implementation must overcome barriers to patient and practice registration and ensure incentives function as intended. Equity is a critical concern: international evidence suggests registration schemes can exacerbate disparities in healthcare access. A recent review found lower registration among socioeconomically disadvantaged populations and those with high healthcare needs (11), with particularly low uptake in migrant and rural communities (18). Lessons from the Australian Health Care Homes trial highlight the importance of community outreach and health literacy (19–21).

Australian practices vary in digital capability, staffing, and support from Primary Health Networks (PHNs), influencing their ability to facilitate registration via the Medicare App or manual processes. Internationally, long waiting lists for registration have been reported in areas with limited providers and high health needs (22), a challenge relevant to rural and remote Australian communities, Aboriginal and Torres Strait Islander peoples, and patients requiring multidisciplinary care.

Evidence from overseas models demonstrates the need to identify benefits to practices to mitigate the risk of perverse incentives such as selective registration of patient cohorts eligible for greater MyMedicare incentives (11). Within the Australian context, current and predicted health workforce shortages (3), particularly among GPs (23, 24) pose additional challenges. Effective implementation also depends on appropriate digital infrastructure and data-sharing mechanisms to enable GP-led care coordination. Linked health and social administrative data are essential for identifying eligible patients and tracking outcomes across primary and hospital care.

### Present study

Previous Australian primary care reforms have been evaluated over short timeframes (12–24 months), limiting insights into changes in service use, costs, and health outcomes. This was evident in evaluations of the Coordinated Care Trials, Diabetes Care Project, and Health Care Homes (7). A longer-term evaluation of MyMedicare is therefore critical to allow sufficient time for registrations to grow, uptake of incentives, and to obtain measurable impacts on CoC and health outcomes. Through an extended evaluation window, the proposed research provides ample opportunity for registration to have occurred at-scale, patients and practices to access incentives and related Medicare item numbers, and for MyMedicare incentives to impact on CoC and health outcomes.

### Aim

The research aims to conduct an early evaluation of the MyMedicare scheme to assess its uptake, implementation, and outcomes by achieving the following objectives:

1. Describe the number and characteristics of accredited GP practices that have registered or withdrawn from MyMedicare.
2. Compare the number and characteristics of patients who have registered, transferred their registration or withdrawn from MyMedicare with characteristics of the Australian population.
3. Explore the implementation of the MyMedicare scheme, including barriers and enablers for registering patients and practices, acceptability, accessibility and costs of registration.
4. Determine whether registered patients experience improved CoC compared to prior to MyMedicare registration and to unregistered patients.
5. Examine health service utilisation of registered patients compared to prior to MyMedicare registration and to unregistered patients.

## Methods

**Figure 1:**
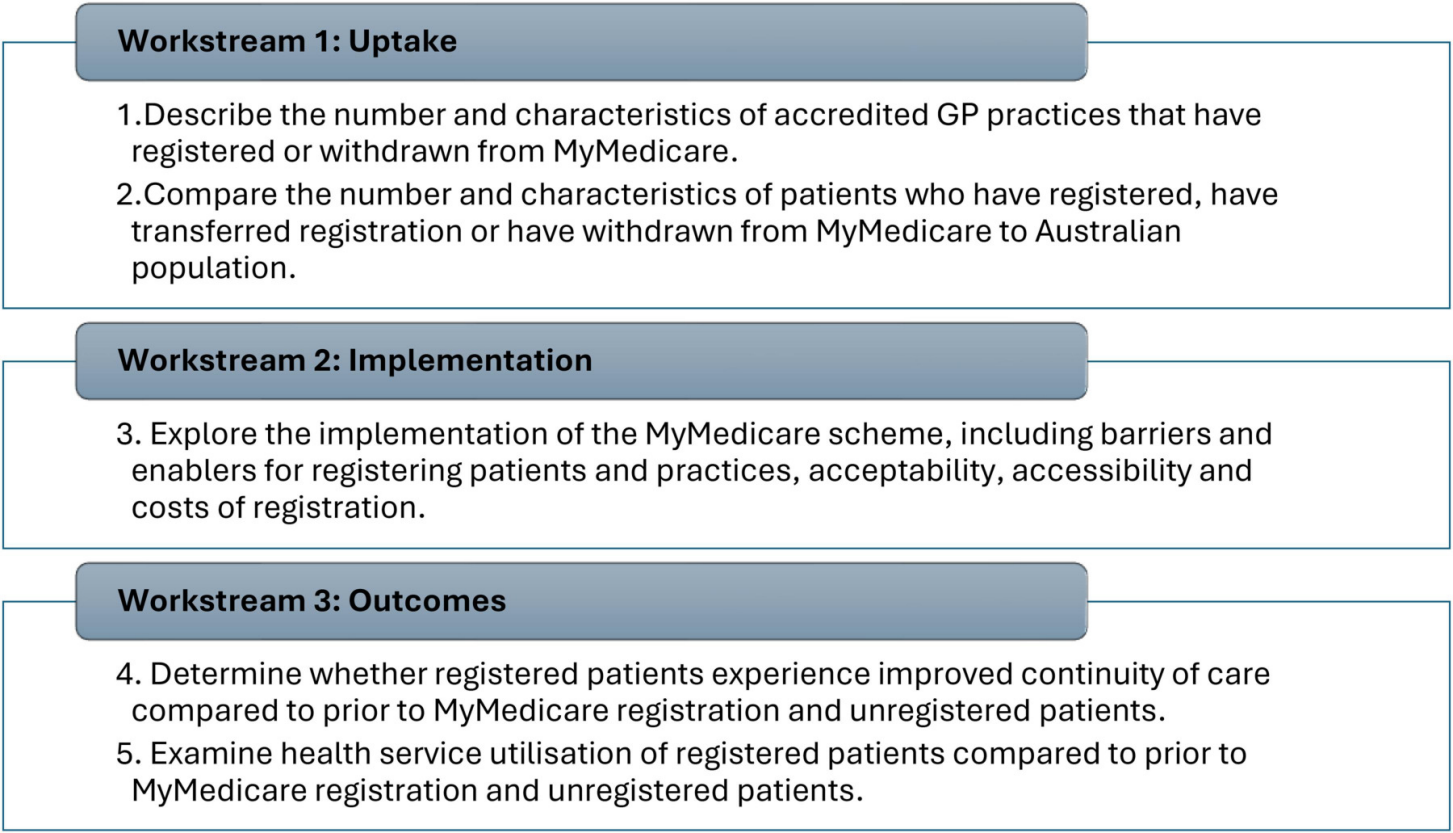
Project overview.

The research comprises three workstreams to address the objectives (Figure 1). Whilst all workstreams will be exploring the experiences, needs and outcomes of Indigenous communities, in accordance with best practice, this component of the research is addressed through a separate ethics approval process following a period of community engagement that is currently underway, supported by the project's Indigenous Advisory Group. This component of the evaluation will therefore be reported in a separate protocol. Further equity considerations are embedded throughout the research in the project team make up, research design, methods and analytic tools. The project was designed by a transdisciplinary team including multidisciplinary clinicians and academics, along with consumers from a range of backgrounds and geographical contexts in Australia. The research approach has been developed in collaboration with more than 10 partners representing all stakeholders. In collaboratively designing the approach, it deliberately captures multi-source information through a variety of data collection methods to capture evidence about the implementation of MyMedicare across the Australian context, including experiences in regional and rural locations, among multicultural communities, people with diverse chronic and complex health needs, and those with diverse gender, sexuality and sex characteristics.

### Workstream 1: uptake of MyMedicare scheme

#### Study design

Descriptive epidemiological study using administrative and publicly available data to examine uptake of MyMedicare nationally to address objectives 1 and 2.

#### Ethical approval

Ethical approval for this study has been provided by the Macquarie University Medical Services HREC, Project ID: 520251943263522.

#### Data sources

Administrative data from Services Australia's Health Professional Online Services (HPOS), publicly available data from the Australian Bureau of Statistics (ABS) 2021 Census data, and data from the National Aged Care Clearinghouse will be used to conduct this study. HPOS includes information on accredited GP practices and providers and Australian patients who are registered with MyMedicare, who have transferred registration to a different practice, or who have withdrawn from MyMedicare registration. ABS Census data includes descriptive information of a snapshot of the Australian population in 2021, including age group, sex, residential location (e.g., urban, regional, remote). National Aged Care Clearinghouse data includes descriptive information on permanent residents within aged care facilities, including age group, sex, and residential location. Comparison of patient population demographics from the HPOS aggregated data to national norms for the Australian and residential aged care populations will be conducted using publicly available ABS Australian Census (2021) and National Aged Care Clearinghouse summary data, respectively.

#### Inclusion criteria

All accredited GP practices and patients registered with MyMedicare in Australia from 1 October 2023 to 30 September 2025, and all GP practices and people in residential aged care who are registered with the GPACI from 1 July 2024 to 30 June 2025 will be included. Accredited GP practices registered with in MyMedicare will be identified via registration status in HPOS.

#### Data analysis

Descriptive analysis will be conducted to identify the number and proportion of registered accredited GP practices for key characteristics, such as region [e.g., primary health networks (PHN) and geographic remoteness]. A descriptive epidemiological profile (e.g., number and percent) of all patients registered with MyMedicare will be produced to report key characteristics, including age group, gender, PHN and geographic remoteness. Demographic characteristics of patients using MyMedicare will be compared (e.g., chi-square tests of independence) with ABS Australian Census 2021 population characteristics and with National Aged Care Clearinghouse characteristics at 2023-24. Where possible, predictors of accredited GP practices and patient registration will be examined for equity considerations using logistic regression, adjusting for covariates (e.g., age group, sex, socioeconomic status, geographic remoteness).

### Workstream 2: implementation of MyMedicare scheme

#### Study design

Mixed methods research using interviews and structured surveys to explore implementation experiences and outcomes in the first two years of the MyMedicare rollout.

#### Ethical approval

Ethical approval for this study has been provided by the Macquarie University Medical Services HREC, Project ID: 520241843160560.

#### Sample

Approximately 80 healthcare staff and 50 consumers (patient, carers and the public) will be recruited. To gather a range of professional experiences from the healthcare workforce, 10 individuals from each of the following eight cohorts will be sought: (1) allied health professionals and pharmacists, (2) Aboriginal health workers, (3) social and support workers, (4) primary care nurses, (5) GPs and other doctors including non-GP specialists, (6) practice staff, (7) PHN staff, and (8) representatives from professional bodies for primary care professions. To gather experiences and perspectives, a minimum of 50 consumers will also be recruited. Sampling will ensure representation of the diverse Australian population in terms of registration status, region, rurality, age, gender, ethnicity, sexuality, health condition and health literacy so that equity considerations and a variety of experiences are reflected in the data.

#### Recruitment

A multi-channel approach will be used through the 10 partner organisations who have agreed to collaborate in this research to inform its direction and support recruitment. The project partners are Carers New South Wales (NSW), National Heart Foundation, LGBTIQ + Health Australia, Australian Hospitals and Healthcare Association, World Wellness Group, Northwestern Melbourne PHN, Pharmaceutical Society of Australia, Arthritis Australia, Rural Doctors Association of Australia and Australian Primary Health Care Nurses Association.

#### Healthcare workforce

Study advertisements will be distributed through partner organisations, PHNs and medical practices nationally. Invitations will also be facilitated by the PHN Cooperative and Practice Based Research Networks that connect research active general practices across Australia. We will recruit a representative group of at least 20 practices that span states and territories, geographic areas, and diverse patient cohorts with whom to explore MyMedicare implementation nationally and recruit staff. Specialists and other health professionals beyond primary care will be recruited via newsletters of our partner professional bodies, via our professional networks and using social media. For practice-based recruitment, we will purposively seek to recruit a sample which approximates the Australian population structure in terms of both jurisdiction and Monash Medical Model.

#### Healthcare consumers

Using our evidence-based recruitment methods, targeted study information has been developed with communities, including easy-read information using images and plain English to support the involvement of consumers with intellectual disability and/or low-English proficiency (25). The study recruitment material for consumers has been developed in collaboration with consumer investigators who are part of the research team. These materials will be shared with research team and partner organisations’ networks to recruit consumers. Purposive sampling will be used to recruit eligible healthcare consumers (18 years or older and eligible for MyMedicare registration) to participate in the semi-structured interviews. This approach will facilitate recruitment of consumers from range of different backgrounds capturing diversity of perspectives and experiences. Healthcare consumers will be reimbursed for any costs incurred and remunerated for their time to participate in the research via gift card based on the amounts recommended in guidance provided by Health Consumers NSW (26).

#### Data collection tools

A topic guide for healthcare staff and another for healthcare consumers has been developed informed by the Theoretical Framework of Acceptability (27), the Consolidated Framework for Implementation Research (CFIR 2.0) (28) and Proctor and colleagues’ Implementation Outcome Taxonomy (29). The topics covered are similar for both cohorts, but plain language is used for consumers and the focus oriented to their health needs. Topics include awareness and knowledge of MyMedicare, its perceived acceptability and relevance, experience of the process of registering or choice not to register, and extent to which MyMedicare has been used in care. A structured cross-sectional online health economic survey has been developed to estimate the costs of patient registration. The survey is to be completed only by GP practices using Research Electronic Data Capture (REDCap).

#### Procedure

Semi-structured interviews of approximately 30–45 min will be conducted by four team members (AC, MP, JM, BN; GA) via telephone, online or in-person. Support needs (such as language support, peer workers) will be established by the research team ahead of the interview and addressed. Interviews will be audio-recorded and transcribed verbatim ahead of analysis. In addition to qualitative data collection, primary care practices and staff will be asked to complete a structured online survey to capture the costs of registering patients, including time taken from other activities. The survey will be completed by practice managers or owners from a range of settings and practice contexts through a REDCap survey link provided by the interviewer at the end of the interview.

#### Data analysis

We will employ a team-based approach to conduct the qualitative data analysis using inductive and deductive methods. Free-text data will be managed using NVivo 14. Inductive analysis (30) will be undertaken by three team members, who will independently code manuscripts line-by-line. Through an iterative process of team-based discussion, themes will be developed that reflect experiences of registration and engagement with patient registration. Themes will be used to describe potential barriers and facilitators to accessing MyMedicare. The framework method (31) will then be used to deductively explore acceptability using the Theoretical Framework of Acceptability which consists of seven components that determine the acceptability of a program or policy: (1) affective attitude, (2) burden, (3) perceived effectiveness, (4) ethicality, (5) intervention coherence, (6) opportunity costs, and (7) self-efficacy (27). The CFIR 2.0 (28) and Proctor et al.'s implementation outcomes (29) will also be embedded in the framework for coding enablers and barriers to implementation (31).

#### Quantitative data

The costs to the GP practices, associated with MyMedicare patient registration, will be estimated by quantifying the time spent by practice staff training, completing administrative tasks, setting up via telephone and online services, and processing registration. Total costs will be calculated as the product of each staff type's hourly wage and the total time they devote to registration activities. Data will be presented separately for GPACI patient registration. The costs will be presented as a median with interquartile range. Variation in costs between practices will be presented descriptively. Calculations will be performed in MS Excel.

### Workstream 3 outcomes of MyMedicare

#### Study design

A series of retrospective population-based controlled pre-post cohort study will be conducted to address objectives 4 and 5: determining whether registered patients experience improved CoC compared to prior to MyMedicare registration and unregistered patients. Due to lack of availability of national level population-based data that includes information on MyMedicare registration status, the Lumos data from New South Wales (NSW), the most populous Australian state will be used to address these objectives (32). MyMedicare registration status will be flagged in the Lumos data from 2026, which will enable this workstream.

#### Ethical approval

Ethical approval for this study has been provided by the Macquarie University Medical Services HREC, Project ID: 520251943263522.

#### Data sources

NSW Ministry of Health's Lumos program (an ethically approved data linkage initiative of NSW Health) provides de-identified data from >900 NSW primary care practices. These data are linked with NSW health service data, including emergency department (ED) presentations, hospital admissions, ambulatory mental health data, NSW Cancer Registry, and mortality data.

#### Inclusion criteria

MyMedicare patients will be identified via MyMedicare registration status within GP practices providing data to Lumos. Individuals who meet relevant inclusion criteria for each study cohort will be identified by the study team using a range of data items within Lumos, including demographic characteristics (e.g., age group, sex, geographic location) and chronic health conditions (e.g., type 2 diabetes, mental health conditions) using SNOMED-CT classifications within the ED datasets and ICD-10-AM diagnosis classifications within hospitalisation data, and MyMedicare registration status. Retrospective data from the estimated 900 primary care provider practices (41%) and up to a third of the 7 million patients in NSW will be included and analysed. A series of pre- and post- MyMedicare registration cohort studies and MyMedicare registrants compared to matched non-registrant comparison studies using the Lumos data will be conducted to examine; (i) number of ED presentations for potentially preventable health conditions (i.e., conditions usually managed in primary care, such as diabetes complications, urinary tract infection);; (ii) number of potentially preventable hospital admissions; (iii) health outcomes, including healthcare use and hospital length of stay, and, where possible, (iv) CoC for people with chronic disease, including cardiovascular disease, chronic obstructive pulmonary disease, type 2 diabetes, mental disorders, and chronic kidney disease based on high burden of disease in Australia.

#### Data analysis

The Lumos linked health datasets will be used to examine CoC and health service outcomes experienced by MyMedicare patients (i.e., cases) identified via MyMedicare registration status and healthcare dates. The pre-period will be defined as 12 months prior to date of the index MyMedicare registration with post-periods at 12 months and then at 24 months post-index registration for cases. The comparison group will be people not registered with MyMedicare during the study period, with 1:1 propensity matched to cases on key patient characteristics of: age group, gender, comorbidities, PHN, geographical remoteness, socioeconomic status, and hospital service use using a greedy algorithm 5-1-digit match (33). Propensity scores will be estimated as the predicted probability of a patient having low (<0.4) CoC compared to medium (0.4–0.7) or high (>0.7) continuity using multinomial logistic regression with stepwise selection of covariates (34). Covariates will be controlled for in statistical analyses and will include: age group, sex, comorbidities (identified using the Charlson comorbidity index using diagnosis and other health records, as appropriate), Indigenous status (Y/N), urban/rural residential location [identified using the Australian Standard Geography Standard (ASGS) and residential postcode or statistical area level 2 (SA2) in health records], hospital length of stay over time (as a proxy for disease progression), disability, ethnicity, and socio-economic status [identified using the Index of Relative Socio-Economic Disadvantage (IRSD) and residential postcode or SA2 in health records].

A series of generalised linear mixed models will be used to examine the association between the number of ED presentations and hospital admissions for MyMedicare patients and usual provider of care (UPC) score, controlling for covariates. A difference-in-differences analysis will compare CoC before and after MyMedicare registration for cases to changes over the same period for matched comparisons not registered with MyMedicare.

## Discussion

This program of work will provide novel, critical evidence of the implementation of the MyMedicare voluntary patient registration scheme, and the associated outcomes within its first years. Conducted over an extended evaluation period, this five-year program of research advances prior evaluative studies of the implementation and impacts of primary care reforms in Australia (6, 7). The proposed research will provide ample opportunity for patients and providers to engage with the MyMedicare scheme to determine its impacts on care. The program of work has been through an independent peer review process as part of the Medical Research Future Fund (MRFF) scheme in Australia; a national competitive grants program. To ensure study quality within the research process, study investigators, including consumers, patient support groups, and health professional bodies meet every three months to review the research progress and make key decisions. Our partner organisations represent diverse patient cohorts, people with a range of health conditions and living in a variety of geographic contexts. Study partners also reflect the diverse range of healthcare professionals impacted by delivering care through the MyMedicare scheme. Individual consumer investigators from a range of cultural, language and geographic backgrounds are also contributing their lived experience to the study process, having been involved since project inception.

Whilst this program of work is funded based on the scientific quality of the proposal, limitations of the available data and context for the research must be acknowledged. As a new scheme, data about MyMedicare registration and date of registration are challenging to obtain and link with national datasets that contain service utilisation and health outcome data. Within the state of NSW, data about MyMedicare registration and registration date are however going to be made available within Lumos and can be utilised to compare service utilisation and outcomes required to achieve the evaluation aims for practices that provide data to Lumos. Yet, Lumos is limited to just over a third of NSW general practices. Patients may attend a Lumos practice and also access care elsewhere in a non-Lumos practice. We cannot be certain therefore that our data about CoC will accurately reflect the entire patient journey and this is a limitation of using these data. If national linked data including MyMedicare registration information becomes available, we will seek access to allow nation-wide evaluation, updating methods accordingly. Using both census and administrative data is an appropriate and robust way to address the questions pose, but we are mindful of the limitations inherent in these datasets, including the potential for missing or incomplete information. When faced with such challenges our approach is to fill missing information in one dataset from other datasets e.g., If age is missing in one dataset, it is often recorded in other datasets. If there are inconsistencies in information across datasets, then we take the most frequent value across all datasets.

Despite the constraints of currently available data, our research will be important in informing policy and practice in Australia and be of value to those in other settings considering voluntary registration schemes. Utilising implementation science frameworks, we will generate rich evidence of the process of VPR implementation, specifically with reference to equitable implementation among the diverse socio-demographic context of the Australian population and unintended consequences. This evidence will demonstrate the support required by communities to engage in the scheme. Our health economic data will provide insight into the costs to practices associated with registering patients, and the financial support required from government to promote the high levels of registration that are needed to achieve the potential gains. In providing evidence and insight to government through regular meetings and feedback sessions, our findings will guide the implementation of MyMedicare and have potential impacts on future health policy relating to the provision of primary healthcare services in Australia.

## Ethics statement

Ethical approval for this research has been provided by the Macquarie University Medical Services HREC, Project ID: 520251943263522.
